# ‘The Jigsaw Culture of Care’: A qualitative analysis of Montessori-Based programming for dementia care in the United Kingdom

**DOI:** 10.1177/14713012211020143

**Published:** 2021-05-23

**Authors:** Shruti Raghuraman, Victoria Tischler

**Affiliations:** European Centre for Environment and Human Health, 3286 University of Exeter Medical School, UK

**Keywords:** dementia, Montessori-Based Programming, montessori methods, dementia care, aged care

## Abstract

**Aims:**

Montessori-Based Programming (MBP) in dementia care refers to a growing body of research and practice that has developed Montessori methods to facilitate self-paced learning, independence and engagement for people living with dementia. A number of research gaps have been identified in the existing literature such as a lack of cross-cultural studies and well-powered, robustly designed outcome studies. The current study investigated the use of MBP with a focus on provision in the United Kingdom. It aimed to identify MBP implementation approaches, challenges and barriers, and research gaps.

**Design and Methods:**

A qualitative design was implemented to analyse data from in-depth, semi-structured interviews with key stakeholders (*N* = 8) with experience of MBP in the UK. Participants included care home management and staff, MBP trainers and independent dementia experts with a background in Montessori methods. Thematic analysis identified 4 main themes and 12 sub-themes. The study took place between April 2019 and October 2019.

**Findings:**

A framework describing knowledge and understanding of MBP in the UK, implementation considerations, challenges and barriers, evidence of outcomes and research gaps was developed to provide guidance for researchers and practitioners. Implementation considerations included using a whole-home approach and changing the culture of care through management support. Barriers to implementation included conservative attitudes to care, perceived lack of time and resources, health and safety issues, and issues of sustainability.

**Conclusion:**

The benefits of MBP in dementia care are promising but require further empirical investigation. There is a need to design, execute and publish evidence to secure the support of key stakeholders in dementia care research, policy and commissioning in the UK.

## Introduction

In the UK, around 850,000 people are living with dementia, of whom at least 70% reside in care homes (Prince et al., 2014). The economic impact of the illness is estimated to be £26.3 billion (£32,250 per person) in the UK, a number which has undoubtedly increased since the publication of this report (Prince et al., 2014). There is an urgent need to explore cost-effective, efficacious interventions for dementia in order to reduce the burden of disease on populations and health systems around the world. There is growing evidence for the potential of non-pharmacological interventions in managing cognitive impairment (Chalfont et al., 2020) and behavioural and psychological symptoms of dementia (Bessey & Walaszek, 2019; Legere et al., 2018). Previous reviews have emphasised the importance of tailoring non-pharmacological interventions to individual needs, preferences and abilities (Bessey & Walaszek, 2019; Scott et al., 2019; Lawrence et al., 2012). This is in line with Kitwood's (1997) foundational work on person-centred dementia care, which emphasises individual agency, dignity and personhood.

### Background

Montessori-Based Programming (MBP) is modelled on the principles of Dr Maria Montessori’s global method of child pedagogy. In the UK, there are approximately 800 Montessori day-care and children’s centres, nurseries and schools, and over 3000 Montessori practitioners (Isaacs, 2018). In the 1990s, the approach was adopted for use in a senior centre in the USA due to its overlap with best practices in dementia care such as tailoring activities relevant to the cultural context and facilitating success over failure (Camp, 2010). MBP emphasises engagement and connectedness alongside the social and physical environment, factors considered crucial for a good quality of life (Malone & Camp, 2007). MBP promotes Kitwood’s concept of ‘rementing’, which is the belief in a person’s continued ability to learn and the possibility of improvements in functioning.

MBP principles include provision of culturally relevant activities, using materials from familiar environments, emphasising structure and order, tailoring activities to personal preference and interest, emphasising invitation and agency, task breakdown, guided repetition, providing cues for self-correcting behaviours and moving from simple to complex, and concrete to abstract tasks (Camp, 2010). Meaningful engagement and enrichment in ‘prepared environments’ that are specially designed for someone with dementia are also integral to MBP (Elliot, 2011). MBP promotes the use of movement and motor learning in purposeful, meaningful activities, as well as using familiar tools to practice and facilitate independent living among people with dementia. The focus is on maximising the individual’s functional, emotional and cognitive abilities to promote fulfilment, enrichment and enhanced well-being (Malone & Camp, 2007). It has also been identified as a non-pharmacological treatment for responsive behaviours (Tschanz & Hammond, 2020), which have been found to be associated with increased caregiver stress and burden (Brodaty & Donkin, 2009). Evidence of MBP’s effectiveness in dementia care is mixed. Research has mostly been conducted in the USA, Canada and Australia. A systematic review of five studies found MBP to be beneficial to people with dementia when compared to routine, control activities such as storytelling, music sessions, socialising, colouring, sensory stimulation and discussion of current events. MBP was found to improve cognitive performance, increase constructive and passive engagement, and enhance affective states (Mahendra et al., 2006). However, the evidence was judged to be of low quality, as well as insufficient to draw definitive conclusions regarding MBP’s efficacy.

Sheppard et al. (2016) reviewed 14 studies that employed MBP in a variety of dementia care settings. Limited evidence of low to moderate methodological quality indicated improvements in eating difficulties and cognition (memory and attention). Mixed evidence was found to indicate improvements in affect and engagement. The authors found several limitations with the evidence base, including a lack of information regarding MBP training and standardised implementation protocols. For the care provider, there is preliminary evidence to suggest that MBP improves staff outcomes such as lower turnover (De Witt-Hoblit et al., 2016) and higher job satisfaction (Booth et al., 2018; Brush et al., 2018).

Hitzig and Sheppard (2017) conducted a scoping review on the implementation of MBP. The most used implementation approach was staff-directed, followed by intergenerational programming (IGP), wherein people with dementia were paired with children under the age of 10 for mutual benefits. Positive outcomes such as increased constructive engagement, reduction in passivity and non-engagement, and reduced responsive behaviours were observed using both approaches. Key benefits for staff included reduced turnover and higher job satisfaction when MBP was staff-directed. The children participating in IGP reported a positive experience overall. Other MBP approaches included resident-assisted programming where those with mild-to-moderate dementia were trained to facilitate MBP for those with advanced symptoms, and family- or volunteer-directed MBP. The authors identified MBP research gaps regarding staff training, economic value and the integration of technology. A lack of evidence from different sociocultural contexts was also highlighted.

Since Hitzig and Sheppard’s (2017) review, we identified six recent publications using the same search terms. Data were extracted and are presented as Supplementary Material. This was used to develop areas of investigation for the current study.

Two independent reviewers extracted the data from these publications to identify updates. We found that staff-directed MBP continues to be the most widely used implementation approach. Studies used different conceptualisations of MBP that were based on similar underlying principles but were branded differently. We also identified a lack of uniformity in defining MBP and how it differs from best practices in dementia care. Importantly, most of the knowledge still originates from the USA, Canada and Australia (Hitzig & Sheppard, 2017; Sheppard et al., 2016).

Despite the growing use of MBP in other countries, there are no published or unpublished accounts of its use in dementia care in the UK. Reviews have highlighted the need to engage global researchers in MBP conversations to facilitate a better understanding of cross-cultural implementation considerations, such as differences in health care systems, knowledge and awareness of MBP, as well as barriers and challenges specific to particular sociocultural or geographical contexts.

This study therefore responds to the lack of research in the UK by investigating the use of MBP with stakeholders who have knowledge of, or currently use MBP in UK care settings. It is anticipated that this knowledge will address the lack of MBP research in the UK and will inform understanding about how MBP may be developed to enhance dementia care.

## Aims

The research questions guiding this study were as follows:

1. What is the current knowledge of MBP in the UK?
2. How is it implemented in care home and community settings?
3. What are the challenges and barriers to its implementation?
4. What are the research gaps in the UK?

## Methods

### Sample/participants

Purposeful sampling was used in line with the qualitative principle of appropriateness, wherein a ‘good’ informant is characterised as one who is articulate, reflective and willing to share expertise with the interviewer (Morse et al., 1991; as cited in Coyne (1997). The sample was selected in the interest of ‘information power’ (Malterud et al., 2016) and obtaining access to ‘information-rich’ participants (Patton, 2002). Individuals with knowledge and expertise of MBP in the UK were identified and invited to participate in the research over email with the aim of gaining detailed information about its use and implementation. Eight individuals were recruited using word-of-mouth and professional networks. The first author and research assistant (RA) contacted the participants by email to explain the purpose of the research and to invite them to take part. The final list of participants included two care home managers; one activities coordinator and one head housekeeper; three independent stakeholders who worked in clinical, long-term care and hospital settings (nursing and occupational therapy); and one independent consultant from a community dementia care setting. All participants self-reported MBP knowledge, training or delivery of 1–2 years duration. The size of the sample was largely determined by pragmatic considerations including availability and access to MBP experts in the UK. Accordingly, we believed we achieved ‘data adequacy’ in accessing the perspectives of senior, experienced MBP trainers, practitioners and stakeholders in the UK (Levitt et al., 2013; Vasileiou et al., 2018). Detailed participant information is presented in Table 1.

**Table 1.** Participant information.

Anonymous ID	Location	Occupation	Years of MBP experience
P1	South East	Independent consultant nurse/MBP trainer	<02 years
P2	South East	Occupational therapist/MBP trainer	<02 years
P3	Midlands	Care home (CH) manager	>01 year
P4	East Midlands	Head housekeeper (CH)	01 year
P5	East Midlands	Activities coordinator (CH)	>01 year
P6	Midlands	Care home manager	>01 year
P7	Midlands	Senior CH manager	02 years
P8	South West	Dementia activist	01 year

MBP: Montessori-Based Programming.

### Data collection

A semi-structured interview schedule was developed, guided by extant literature in the field, particularly previously published reviews (Hitzig & Sheppard, 2017; Mahendra et al., 2006; Sheppard et al., 2016). Topics included participants’ understanding of the Montessori method, its adaptation for use with people with dementia, benefits of MBP, and challenges, barriers, and facilitators to implementation and training. The RA conducted one-to-one interviews with each participant. Interviews were audiotaped following voluntary, written consent and were transcribed verbatim. The interviews lasted for an average of 54.09 min.

### Ethical considerations

Ethical approval for this research was obtained from the Research Ethics Panel, College of Nursing, Midwifery and Healthcare, University of West London (Ethical Approval No. 00680). All participants provided written informed consent before partaking in the interviews.

### Data analysis

The transcripts were analysed using thematic analysis (TA), guided by the six-phase method (Braun & Clarke, 2006). TA is not wedded to a specific, pre-existing theoretical framework, which makes it apt for a foundational study of this nature where knowledge is still developing. As little is known about MBP in the UK, the analysis was primarily inductive while also being guided by the literature review.

The six phases of the analysis are as follows: (a) familiarising oneself with the data, (b) generating initial codes, (c) searching for themes, (d) reviewing themes, (e) defining and naming themes and (f) producing the report. A detailed description of each phase is included as Supplementary Material.

The data were coded in the first instance by the RA who developed an initial coding framework. This was checked against the transcripts by a second analyst to allow for alternative interpretations. Comparisons and disagreements were resolved, and the coding framework was refined over the course of three meetings to develop the findings.

## Findings

A multi-faceted framework of MBP in the UK was derived from the analysis of the interviews and is presented in Table 2. The four themes presented focus on MBP understanding, implementation, challenges and barriers and research gaps in the UK context. These are presented below, using sub-themes to elucidate, supported by illustrative quotes from participants.

**Table 2.** Thematic framework – MBP in the UK.

Theme	Subtheme
What is MBP?	(1) Multidisciplinary approach
	(2) Approach versus activities
Implementation considerations	(1) Changing culture – ‘Top down’
	(2) Whole-home approach
	(3) A marathon, not a sprint
Challenges and barriers	(1) Novelty and conservatism
	(2) Time and resources
	(3) Sustainability
	(4) Health and safety
Research gaps and the way forward	(1) Understanding MBP
	(2) Evidence-base
	(3) Training and dissemination

MBP: Montessori-Based Programming.

### What is MBP?

#### Multidisciplinary approach

The use of MBP is in its nascent stages, especially in the UK. As a result, its application and understanding in the UK cultural context was particularly important to participants.

The participants’ indicated that MBP is a multidisciplinary approach, with an amalgamation of several professional approaches required to implement it effectively. This included speech and language therapy, psychology, nursing, occupational therapy and education. As P2 stated:

*‘It's bringing everything together. I think Montessori is the golden thread that that brings it all together. For a speech and language therapist, it is around signage. But I think you have to take a bit of that, a bit of occupational therapy, a bit of psychology and a bit of everything else. Put it together. Now comes magic.’*

#### Approach versus activities

The participants differentiated the MBP approach from the execution of Montessori activities. According to participants, MBP does not dictate what to do, but how to do it. P1 said:

*‘It's an approach, you see. So, it's not an activity and one of the things we have to stop people saying is “I'm going to do a Montessori activity”... the way that you engage people is Montessori.’*

Some of the underlying principles of MBP discussed by participants overlapped with person-centredness (Kitwood, 1997) and included tailoring activities to personal interests, preferences and background, embedding activities in the specific cultural context of the individual, and the notions of living with purpose, dignity and respect. As P2 stated:

*‘It's knowing that person. Going back to the lady [resident with dementia] who wanted to sweep the kitchen, she was happy sweeping, because that’s been her job before, when she was a teenager.’*

Communicative practices such as invitation and offering choice were identified as key to MBP. These were in line with Montessori philosophy as highlighted by P3:

*‘It's offering people choice. … people think oh, no, they [people with dementia] can't, they can't take the choice. But they can if you give them the right cues, they can make the choice.’*

Enabling independence and focusing on strengths and abilities was found to be fundamental to MBP. P2 spoke of her experience with a couple in the community setting:

*‘He [carer] had taken away every task that she [person with dementia] had ever done. He was cooking, he was helping her get dressed. He didn't want to do those sorts of things. So, we unpicked a little bit of it. I just put some labels up. Before that he was having to get all her clothes out but with the labelling and giving her the cueing, she could find what she needed to do.’*

The ‘prepared environment’ is an important aspect of MBP in dementia care (Elliot, 2011). This includes elements of task breakdown, guided repetition, signage, and cueing the individual to tasks and activities, all of which enable independence and failure-free engagement. P2 discussed this:

*‘If you want to develop an activity with them, it's not having the clutter around. It’s having that clean environment that you can work in - the very prepared environment. It's about cueing them into looking at that activity and what you're trying to do.’*

### Implementation considerations

#### Changing culture – ‘Top down’

A culture change was seen as important to implementation, particularly in care homes. This was contrasted with traditional approaches to care that were considered outdated and redundant, for example, P7 stated:

*‘People [care home staff] that have potentially been around perhaps a long time have become very task-orientated in that, “it's eight o'clock, it's bath time” or “it's Tuesday, let's have a bath.”’*

Participants highlighted the tradition of doing things for the person with dementia as being ‘degenerative’. MBP emphasises enabling activity and independence, emphasising strengths and abilities. It was also suggested that empowering staff to act independently, take ownership and solve problems was crucial to MBP. As P7 stated:

*‘I'd like to see the culture really embedded, that when I go into the home, it will be just full of residents doing what they want, when they want, with staff supporting them, and actually [staff] start feeling okay and comfortable with it.’*

As is true of any setting, a culture change is rarely possible without commitment from management. This is especially relevant in care homes, where participants attributed success and failure of MBP implementation to vision, leadership and support from the management. As P3 discussed:

*‘It's got to come from the top down. You've got to have support from your senior managers and the directors, and then it has to filter down through to keep the momentum going.’*

#### Whole-home approach

Participants repeatedly discussed the importance of cooperation and involvement of all those in the setting in which MBP was being used. By ‘whole-home approach’, participants referred to the training and development of all staff as opposed to restricting it to care staff and activity coordinators. According to P1:

*‘[the key to successful implementation] is that the care home has taken a whole-home approach, and the company has taken a whole-home approach. And that is absolutely vital, because that is the only way you're going to change culture and practice.’*

This is also true of families who provide care in their homes. Without the consensus of every family member, participants indicated that successful implementation of MBP was impossible. As P2 shared:

*‘You may have one daughter or one son that allows them [person with dementia] to take those risks, and others that want them to be totally safe and put in a care home. You know, that's it, that's a barrier.’*

Participants stressed the need to challenge the traditional ‘pyramid culture of care’, which emphasises hierarchy and focuses on care staff. P1 said:

*‘Instead of having the traditional pyramid culture, where the hierarchy is one of the biggest issues, and where the bottom is the kitchen staff, and the top is the manager, we emphasise the jigsaw culture of care, where everybody is equal.’*

#### A marathon, not a sprint

Enthusiasm for MBP was evident, with participants speaking highly of the drive and motivation they experienced once they were acquainted with the approach. However, any changes and modifications to routine care had to be implemented incrementally to ensure success. P7, a care home manager, cautioned:

*‘There's no good saying to them [staff], well, the residents are going to go into the laundry to do the laundry. Instead, we start with, “Can you get the residents to fold some of the towels” and then we'll take it a step or two steps at a time.’*

### Challenges and barriers

#### Novelty and conservatism

MBP is a relatively new care concept in the UK. When questioned about potential barriers to its implementation, participants highlighted the lack of knowledge and understanding of MBP as a key concern. P2 suggested:

*‘I don't think anybody knows about it. I don't think that is out there. The challenge is getting people fully educated in what Montessori means.’*

A further concern is that a lot of people are not aware of its application to dementia care. There is a risk that it may be seen as infantilizing people with dementia. According to P6:

*‘A barrier is people not understanding it, because some people think it is for children. But it is the right environment for people with dementia as well.’*

Traditional approaches to care dominate and may continue to serve as a barrier to changing the culture of dementia care. In care home settings, staff who struggle with change may threaten successful implementation. As P1 said:

*‘And you will get that that resistance, and dynamic conservatism as it used to be called where people will not change, because “I'm not going to be told, I've been doing this job for 20 years…”’*

#### Time and resources

Management may face a lack of resources when trying to motivate employees to adopt MBP. However, some indicated that, in the longer term, MBP could save time. As P3 noted:

*‘We’re faced with the response, “we don't think we've got time to do all this, we’re so busy doing x y & z.” But if you spend a little bit more time helping the residents to help themselves, you'll find you've got much more time… then they're spending less time actually dealing with somebody that's agitated.’*

Other barriers included the time required to build a relationship with the person with dementia to ensure meaningful engagement and positive outcomes. P8 advised:

*‘It's about building trust. You can't be superficial; you're not going to get that engagement with someone if they don't feel comfortable’.*

#### Sustainability

Stakeholders in both community and care home settings raised the issue of how to make MBP sustainable. As P2 stated:

*‘We can go in, we can teach, we can start it. But it has to be sustained by the organisation. Or if you're talking about the community, it is still training or teaching people the philosophy and keeping up that understanding’.*

#### Health and safety

Enabling activity and independence for the person with dementia involves encouraging the individual to extend their physical and mental capacities to participate in meaningful tasks. However, this poses potential threats to health and safety, especially in care homes where risk assessments are mandatory. P3 raised this as a challenge:

*‘We weren't allowed to let people take risks because what if they hurt themselves when peeling the potatoes? We all got bogged down with health and safety.’*

### Research gaps and the way forward

#### Understanding MBP

MBP is very much in its infancy in the UK, with stakeholders finding it challenging to further develop their practice. This is partly due to confusion over how MBP differs from other models of good practice. As P2 said:

*‘[Initially] I found [MBP] quite chaotic, to try and understand the Montessori principles and all the other professional principles around nursing, speech and language therapy’*

However, they also went on to articulate their understanding of MBP over time:

*‘Now I've got more knowledge…I'm talking about the courtesy, the grace and the prepared environment. And those things, those I see [are] the key Montessori bits that we've taken and wrapped everything else around.’*

#### Evidence-base

A lack of understanding of MBP in the UK can be attributed to the scarcity of research to date. Much of the evidence-base for MBP comes from the USA, Canada and Australia (Hitzig & Sheppard, 2017). As P1 stated:

*‘So, until we can get some academically rigorous evidence to show what it's doing for individuals, it will still be considered a fad, or a fancy, really. And that is the challenge.’*

Further research to identify the potential benefits of implementing MBP in dementia care settings will create academic interest in the topic and should contribute to a better understanding of MBP in different global contexts. It will also support development of training programmes, which are central to improving the care culture in the UK.

#### Training and dissemination

Differences in care home cultures around the world were highlighted as important to consider in adapting the approach for global dissemination. P3 talked about the importance of culturally relevant materials,

*‘What we saw from the homes in Australia, is that they are very different to those in in England… [what we need is] to actually take the evidence from care homes that are familiar to us.’*

P7 was a recipient of MBP training and shared their first-hand experience of how the learning material must be bespoke to the trainees. For example,

*‘[we need] structured training, that is [at] an acceptable level, because you know, when you're, you're looking at people [whose] education is different. It needs to be practical so that everybody gets it.’*

## Discussion

This study was the first to explore the use of MBP in the UK. The results identified good practice in implementing MBP in care homes; however, there was generally a lack of knowledge, resources and support for the approach that signified a barrier to further development and use in the UK. The findings were used to develop a detailed thematic framework to map current use that should guide future researchers and practitioners on the study and implementation of MBP in the UK.

Knowledge and understanding of MBP was found to be aligned with Montessori methods (Lillard, 2005) and global conceptualisations of MBP (Camp, 2010; Elliot, 2011), which are also in line with best practices in dementia care. Examples include tailoring activities based on personal interests, preferences, and background; embedding activities in an individual’s cultural context; and the notions of purpose, dignity and respect. The importance of invitation, choice, task demonstration and breakdown were also identified in line with Montessori philosophy.

Of key concern is the lack of research evidence for MBP in the UK including outcomes and benefits. The need to engage the interest of researchers in designing rigorous studies that build on anecdotal evidence was identified in our findings. This was also raised as a knowledge gap by a previous review, which called for randomised controlled trials to measure outcomes of MBP in care settings (Hitzig & Sheppard, 2017).

This is linked to an implementation barrier in the UK. Participants cited the lack of MBP knowledge, understanding and evidence, along with the fear of perceived infantilization of care recipients. This was reflected in a Canadian qualitative study which discussed the importance of educating staff and families as crucial to preventing the perception of infantilization when using MBP (Ducak et al., 2018). Our study revealed the need to educate health and social care professionals to ensure their support in the implementation of MBP in dementia care.

Similar to Ducak et al. (2018), medical models of care which emphasise task-orientated practices and a pyramid culture that emphasises staff hierarchy were found to be barriers to MBP implementation. Participants in our study felt that this culture prevents non-care staff getting involved in adopting MBP in their interactions with care recipients, which impedes change. Culture change was identified as a crucial implementation consideration and was linked to empowering carers and non-care staff to adopt MBP. This included engaging residents in activities that are personalised and meaningful, using tasks related to their previous roles, ensuring their interests and preferences are taken into account and using time and resources as efficiently as possible to maximise outcomes. This is related to the participants’ view that enabling residents to be more active and engaged could be mutually beneficial to the care home management. Studies have demonstrated the success of resident-assisted Montessori programming in care home settings in improving active engagement and pleasure for residents (Camp et al., 2005; Camp & Skrajner, 2004; Skrajner et al., 2014; Skrajner & Camp, 2007). The findings from this study demonstrate that implementing the Montessori approach could uncover the potential of residents to assist in the care of their peers in the UK context.

Ongoing, sustained support from management was also emphasised as critical to sustain MBP practice. The importance of management support in embedding a Montessori culture of care has been similarly stressed in previous research (Bourgeois et al., 2015; Ducak et al., 2018). This raises the challenge of sustainability of MBP in care settings as participants reported that staff can struggle to support provision that promotes independence and meaningful engagement and enables dignity and purpose for the person with dementia. This is associated with issues of limited time and resources to implement new approaches to care, which are often raised as concerns when carers, especially in formal care settings, implement MBP. However, participants indicated that culture change can positively impact resources.

In their review, Hitzig and Sheppard (2017) emphasised a need for structured and bespoke training, which was reflected by participants in our study. Additionally, we found that for the training programme to be meaningful, it had to be tailored to the cultural context, for example, UK care settings.

Importantly, training and engaging the whole home (or the entire family in community settings) was key to successful implementation of MBP. Some instances of training only skeletal care staff did not provide sustained changes to the culture of care, but when all staff were included, the effects were profound. This is linked to educating all parties who may be directly or indirectly involved in care delivery, for example, domestic staff and carers to maximise benefits.

It remains unclear how MBP is conceptualised in the UK and how it is different from other recommended practices such as person-centred care. This was one of the key research questions; however, our findings suggest that MBP is still poorly defined and understood within dementia care. One of the challenges appears to be the prevalence of multiple trademarked Montessori approaches in different countries such as Montessori-Based Dementia Programming^®^ (MBDP^®^) (Malone & Camp, 2007), Montessori Methods for Dementia^TM^ (Elliot, 2011) and DementiAbility Methods: The Montessori Way™ (O’Neill et al., 2014). These programmes, although sharing foundational principles, offer separate workshops and training in MBP. This contributes to the lack of a homogeneous conceptualisation of MBP, which influences our findings. This remains a research and knowledge gap and must be addressed in future studies.

While the current body of knowledge on MBP is incomplete, there is evidence of potential benefits for care home residents and staff (Cartwright & Oliver, 2015; Mahendra et al., 2006; Sheppard et al., 2016). The data from this study support this and indicate the need to further investigate, adapt and implement MBP in the UK to replicate the benefits found in outcome studies from other countries.

### Limitations

The study sought to gain insight into MBP knowledge and implementation from stakeholders who had expertise in developing, delivering or training in MBP. We did not include the views of family carers, which could provide additional perspectives regarding MBP provision in formal and informal care settings.

Further, some participants were employed in the same care home franchise and had been trained by a team whose views are also represented in this study. This may have influenced their observations and understanding of MBP. One participant, who had been trained by a different provider, had experience in community implementation of MBP thus representing an alternative perspective.

Future research should engage more community and non-professional carer perspectives, as well as lived experiences of people with dementia to gain a more holistic understanding of MBP adaptation and implementation in the UK.

## Conclusion

There is an urgent need to identify and evaluate cost-effective, non-pharmacological approaches to dementia care that are efficacious in improving quality of life as well as alleviating responsive behaviours, non-engagement, physiological deterioration and overreliance on carers. There is initial evidence from various Western settings to suggest that MBP can produce positive outcomes and manage negative outcomes for people with dementia, such as responsive behaviours and improve staff outcomes such as enhanced job satisfaction. Yet, there remain gaps in knowledge and research regarding a uniform understanding of MBP, its processes, attributable outcomes, economic value, training and development. There is a lack of discussion around the barriers and challenges to implementation of MBP, which is crucial to support dissemination to families, care practitioners, organisations and policymakers. It is anticipated that the findings from this study of MBP in the UK will support further research efforts. This should lead to the development of culturally relevant MBP, including training and implementation to guide future practice in the UK and globally.

## Supplemental Material

sj-pdf-1-dem-10.1177_14713012211020143 – Supplemental Material for ‘The Jigsaw Culture of Care’: A qualitative analysis of montessori-based programming for dementia care in the United Kingdom Click here for additional data file. Supplemental Material, sj-pdf-1-dem-10.1177_14713012211020143 for ‘The Jigsaw Culture of Care’: A qualitative analysis of montessori-based programming for dementia care in the United Kingdom by Shruti Raghuraman and Victoria Tischler in Dementia

sj-pdf-2-dem-10.1177_14713012211020143 – Supplemental Material for ‘The Jigsaw Culture of Care’: A qualitative analysis of montessori-based programming for dementia care in the United Kingdom Click here for additional data file. Supplemental Material, sj-pdf-2-dem-10.1177_14713012211020143 for ‘The Jigsaw Culture of Care’: A qualitative analysis of montessori-based programming for dementia care in the United Kingdom by Shruti Raghuraman and Victoria Tischler in Dementia
