# Supplementary material for: ‘The Jigsaw Culture of Care’: A qualitative analysis of Montessori-Based programming for dementia care in the United Kingdom
Source: Dementia (London). 2021 May 23;20(8):2876–90. doi: 10.1177/14713012211020143 (PMC8678648; doi:10.1177/14713012211020143)
Supplement: sj-pdf-1-dem-10.1177_14713012211020143 – Supplemental Material for ‘The Jigsaw Culture of Care’: A qualitative analysis of montessori-based programming for dementia care in the United Kingdom [file sj-pdf-1-dem-10.1177_14713012211020143.pdf]

Supplementary file 1. Review of recent MBP research

**1**	**Hunter et al., 2018**<br><br>**Location:** Canada	**Design:** Qualitative analysis<br><br>**Sample:** Care home staff (N=21)<br><br>**Approach:** Volunteer-directed	**Aim:** To identify process of integrating Montessori-based intervention into practice and explore staff perceptions of strengths and weaknesses<br><br>**MBP Intervention:**<br>30x activity boxes; 4 domains - practical; sense stimulating; culturally meaningful; cognitively stimulation. Researchers identified suitable boxes for each participating resident	**Overall findings**<br>Activity kits tailor made and tested on residents to find the best fit based on residents' skills, abilities and disabilities, background, and interests<br><br>**Strengths:**<br>- Positive overall staff perceptions on the intervention<br>- Positive outcomes on residents on outcomes of interpersonal relationships, mood/behavioural outcomes, meaningful occupations<br>- Positive staff outcomes on efficiency<br>- Volunteers fitted in well<br>**Weaknesses:**<br>- Extensive recruitment and training required<br>- Risk management<br>- Limited benefits for advanced dementia
**2**	**Douglas et al., 2018**	**Design:** Case study<br><br>**Sample:** NA	**Aim:** To provide practical strategies to support implementation of Montessori into SLP practice in according to person-centred approaches	**Overall findings**<br>**1. Environment:**<br>- Montessori classroom components<br>- Sensory cues<br>- High contrast signage<br>- Importance of personalization of spaces

	**Location:** USA	**Approach:** Staff-directed	**MBP Intervention:** Montessori activities suggested are broad and intended as part of SLP interventions, supported by care home staff. Examples given include laundry, book reading, table setting.	**2. Care for self:**<br>- Independence in self-care emphasized for dignity and self-respect<br>- Importance of invitation by facilitators to build routine & spontaneity<br>- Match cognitive abilities & interests<br>**3. Care for others**<br>- Meaningful relationships for increased well-being
**3**	**Wilkes et al., 2019**	**Design:** Intervention study; interrupted time-series design<br><br>**Sample:** Residents with advanced dementia (N=43)	**Aim:** To evaluate the effects of a Montessori-based activity program implemented in a long-term care facility	**MOBA Embedding:** Two-phase process where MOBA instructors worked beside staff to embed programming and principles learned in prior training sessions. A lead social worker was selected to oversee all programming.

	**Location:** USA	**Approach:** Staff-directed; trained nursing/clinical staff, care workers, activity leaders, kitchen staff, management	**Intervention: 'MOBA'**<br>- Customised lesson planning (based on interests and capabilities, ability to ensure success); Intellectually stimulating exercises; Sensory puzzles & games; Group readings; Engagement in household activities;<br>- Immediate feedback, right to refuse + provided with alternatives<br>**-** Duration: 7x/week; 8 months	**Overall findings**<br>- Moderately reduced problem behaviours<br>- Significantly decreased social engagement and activities of daily living capacities<br>- Quality of life increased initially but diminished in the final period<br>- Anxiety and well-being decreased initially but elevated in the final period
**4**	**Yuen et al., 2018**	**Design:** Randomised-controlled trial; Control condition - structured social activities<br><br>**Sample:** Residents with moderate to severe cognitive decline and significant agitation (N=46)	**Aim:** To evaluate the effects of a Montessori-based activity program implemented in a long-term care facility	**Overall findings**<br>- Significantly reduced the frequency and disruptiveness of overall agitation to greater extent.<br>- Effective in reducing agitation in long term care home residents with dementia compared to controls<br>- Preference of local clinicians lie with Montessori intervention
	**Location:** Hong Kong	**Approach:** Staff-directed; trained	**MBP Intervention: 'DMMW'**<br>- 5 components: 1) Invitation, 2)	

		nursing/clinical staff, care workers, activity leaders, kitchen staff, management	demonstration, 3) Needs, 4) Therapeutic Environment, 5) Extension and conclusion<br>**-** Duration: 3x/week, 6 sessions,	
**5**	**Booth et al., 2018**<br><br>**Location:** Australia	**Design:** Qualitative; thematic analysis<br><br>**Sample:** Staff (N=4)<br><br>**Approach:** Staff-directed	**Aim:** To examine whether Positive Interactive Engagement (PIE) programme reduces challenging behaviours associated with 'sundowners' syndrome<br><br>**MBP Intervention: 'PIE'**<br>- Activities designed to enhance connection and resident interest by focusing on engaging in pleasing activities and using everyday materials<br>- No specialist equipment<br>- Physical and gross motor skills	**Overall findings**<br>**-** Reduced disruptive behaviours, positive behaviours remained the same/increased;<br>- Increased social connection<br>- Increased job satisfaction among staff<br>- Routine (structure, content, timing & familiarity) key factors to success<br>- Individualised nature of activities (flexibility, individual attention) key to success<br>- Strength lies in combined approach of four components - a) sensory activities, b) high, c) low intensity activities and d) relaxation

			emphasised<br>- Fluid and responsive to continuously changing cohort of residents<br>**-** Duration: Daily, 7 total sessions	
**6**	**Mbakile-Mahlanza et al., 2019**<br><br>**Location:** Australia	**Design:**<br>Cluster randomized crossover design<br>Control condition: Psychoeducation and newspaper reading<br><br>**Sample:** Resident-caregiver dyads (N=20 dyads)<br><br>**Approach:** Carer-directed<br>Carers trained in theoretical and individualised, practical approach to Montessori-based care	**Aim:** To evaluate the impact of the Montessori activities implemented by family members on visitation experiences with people who have dementia.<br><br>**MBP Intervention:**<br>**-** Activities chosen by trained family carers based on residents' former interests and current language and motor skills<br>- Residents interact with carers while engaging in activities.<br>- Duration: 2x/week, 2 weeks	**Overall findings**<br>**- Resident outcomes**<br>**--** Affect (pleasure, anger, anxiety, and contentment) and engagement<br>--Significant within-group effects in the Montessori condition<br>--Significant between-group effects in favour of the Montessori condition<br><br>**- Caregiver outcomes**<br>--Higher visit satisfaction, higher relationship quality & higher depressed mood in the Montessori condition compared to controls<br>**--** Promising results for family carer-led intervention, especially in terms of individualisation of activities
