# Supplementary material for: ‘The Jigsaw Culture of Care’: A qualitative analysis of Montessori-Based programming for dementia care in the United Kingdom
Source: Dementia (London). 2021 May 23;20(8):2876–90. doi: 10.1177/14713012211020143 (PMC8678648; doi:10.1177/14713012211020143)
Supplement: sj-pdf-2-dem-10.1177_14713012211020143 – Supplemental Material for ‘The Jigsaw Culture of Care’: A qualitative analysis of montessori-based programming for dementia care in the United Kingdom [file sj-pdf-2-dem-10.1177_14713012211020143.pdf]

Supplementary file 2. Phases of thematic analysis (Braun & Clarke, 2006; p. 87)

Phase	Description of the process
1. Familiarizing yourself with your data:	Transcribing data (if necessary), reading and re-reading the data, noting down initial ideas.
2. Generating initial codes:	Coding interesting features of the data in a systematic fashion across the entire data set, collating data relevant to each code.
3. Searching for themes:	Collating codes into potential themes, gathering all data relevant to each potential theme.
4. Reviewing themes:	Checking if the themes work in relation to the coded extracts (Level 1) and the entire data set (Level 2), generating a thematic 'map' of the analysis.
5. Defining and naming themes:	Ongoing analysis to refine the specifics of each theme, and the overall story the analysis tells, generating clear definitions and names for each theme.
6. Producing the report:	The final opportunity for analysis. Selection of vivid, compelling extract examples, final analysis of selected extracts, relating back of the analysis to the research question and literature, producing a scholarly report of the analysis
